# Diagnostic significance of hsa_circ_0000146 and hsa_circ_0000072 biomarkers for Diabetic Kidney Disease in patients with type 2 diabetes mellitus

**DOI:** 10.5937/jomb0-39361

**Published:** 2023-03-15

**Authors:** Amul M. Badr, Omayma Elkholy, Mona Said, Sally A. Fahim, Mohamed El-Khatib, Dina Sabry, Radwa M. Gaber

**Affiliations:** 1 Cairo University, Faculty of Medicine, Medical Biochemistry and Molecular Biology Department, Cairo, Egypt; 2 Newgiza University (NGU), School of Pharmacy, Department of Biochemistry, Newgiza, Giza, Egypt; 3 Cairo University, Kasr El-Aini Medical School, Department of Internal Medicine, Nephrology Section, Cairo, Egypt; 4 Badr University in Cairo, Faculty of Medicine, Medical Biochemistry and Molecular Biology Department, Badr city, Egypt

**Keywords:** diabetic kidney disease, cannabinoid receptor 1, miR-21, miR-495, circ_0000146, circ_0000072, dijabetesna bolest bubrega, kanabinoidni receptor 1, miR-21, miR-495, circ_0000146, circ_0000072

## Abstract

**Background:**

Diabetic Kidney Disease (DKD) is a significant challenge in healthcare. However, there are currently no reliable biomarkers for renal impairment diagnosis, prognosis, or staging in DKD patients. CircRNAs and microRNAs have emerged as noninvasive and efficient biomarkers.

**Methods:**

We explored Cannabinoid receptor 1 (CNR1), C reactive protein (CRP), hsa_circ_ 0000146 and 0000072, and hsa-miR-21 and 495 as diagnostic biomarkers in DKD. The serum concentrations of CRP and CNR1 were measured using ELISA. Rt-qPCR was used to evaluate the expression levels of CNR1, circRNAs, and miRNAs in 55 controls, 55 type 2 diabetes mellitus patients, and 55 DKD patients. Their diagnostic value was determined by their ROC curve. KEGG pathway was used to predict the functional mechanism of the circRNA's target genes.

**Results:**

DKD patients exhibited a significant increase in CRP and CNR1 levels and the expression of miR-21 and 495. The expression levels of circ_0000146 and 0000072 decreased in DKD patients. ROC analysis revealed that circRNAs and miRNAs alone or CNR1 and CRP have significant diagnostic potential. The functional prediction results showed the involvement of hsa_circ_0000146 and 0000072 in various pathways that regulate DKD.

**Conclusions:**

Therefore, the examined circRNAs and miRNAs may represent a novel noninvasive biomarker for diagnosing and staging DKD.

## Abstract (sr)

**Uvod:**

Dibetesna bolest bubrega (DKD) predstavlja značajan izazov u zdravstvu. Međutim, trenutno ne postoje pouzdani biomarkeri za dijagnozu, prognozu ili određivanje stadijuma oštećenja bubrega kod pacijenata sa DKD. CircRNA i mikroRNA su se pojavili kao neinvazivni i efikasni biomarkeri.

**Metode:**

Istražili smo kanabinoidni receptor 1 (CNR1), C reaktivni protein (CRP), hsa_circ_ 0000146 i 0000072, i hsa-miR-21 i 495 kao dijagnostičke biomarkere za DKD. Koncentracije CRP i CNR1 u serumu su merene korišćenjem ELISA. Rt-kPCR je korišćen za procenu nivoa ekspresije CNR1, circRNA i miRNA kod 55 kontrolnih pacijenata, 55 pacijenata sa dijabetes melitusom tipa 2 i 55 pacijenata sa DKD. Njihova dijagnostička vrednost određena je njihovom ROC krivom. Za predviđanje funkcionalnog mehanizma ciljnih gena circRNA korišćena je KEGG mapa puteva.

**Rezultati:**

Pacijenti sa DKD su pokazali značajno povećanje nivoa CRP i CNR1 i ekspresije miR-21 i 495. Nivoi ekspresije circ_0000146 i 0000072 su se smanjili kod pacijenata sa DKD. ROC analiza je otkrila da samo circRNA i miRNA ili CNR1 i CRP imaju značajan dijagnostički potencijal. Rezultati funkcionalnog predviđanja pokazali su učešće hsa_circ_0000146 i 0000072 u različitim putevima koji regulišu DKD.

**Zaključak:**

Stoga, ispitivani circRNA i miRNA mogu predstavljati novi neinvazivni biomarker za dijagnostikovanje i određivanje stadijuma DKD.

## Introduction

Diabetic kidney disease (DKD) is a significant condition that affects up to 50% of people with diabetes. It develops into end-stage kidney disease (ESKD) [1]. The gold standard for diagnosing DKD is microalbuminuria, glomerular filtration rate (GFR) based on creatinine or cystatin C, and kidney histology [2]. These diagnostic markers are unreliable, insensitive, costly, and invasive. The lack of cost-effective, reproducible, and noninvasive biomarkers for DKD is the leading cause of delayed diagnosis and treatment. Consequently, there is growing interest in developing alternative prognostic or predictive biomarkers.

The endocannabinoid system comprises type 1 (CNR1) and type 2 (CNR2) cannabinoid receptors and ligands that are dominant in the kidney [3]. In DKD, CNR1 signaling contributes to the formation of inflammation and fibrosis [4]. C reactive protein (CRP) is an acute-phase inflammatory protein linked to microalbuminuria and renal impairment in T2DM patients [5].

Noncoding RNAs (ncRNAs), miRNAs, and circRNAs are epigenetic regulators. miRNAs are single-stranded, small (19–25 nucleotides) noncoding RNAs that have recently gained prominence as vital regulators of gene expression [6]. hsa-miR-495 has been linked to several immunological and inflammatory processes, cancer cell proliferation, metastasis, and treatment resistance [7]. Multiple gene regulatory functions of hsa-miR-21 are associated with complications of T2DM, and its silencing ameliorates DKD [8]. CircRNAs are covalently closed RNA loop products generated by back-splicing during transcription. They act by sponging miRNAs and proteins that regulate their expression [9]. CircRNAs are additionally distinguished by being structurally stable and tissue- specific [10]. Intriguingly, studies hypothesize that circRNAs regulate the inflammation and fibrosis of the proximal tubules caused by high glucose levels [11]. Due to the paucity of information on circRNAs and DKD, bioinformatics analyses were used to select circ_ 0000146 and 0000072, which serve as sponges for miR-21 and miR-495, respectively.

Therefore, this study examines expression profiles for CNR1, CRP, hsa_circ_0000146 and 0000072, and hsa-miR-21 and 495 as potential noninvasive biomarkers for the diagnosis of DKD.

## Materials and methods

### Subjects

A case-control study (approval number: MD-83-2020) was conducted at the Medical Biochemistry and Molecular Biology Department of Cairo University’s Faculty of Medicine. All participants gave their informed consent for this study, which was carried out in conformity with the Declaration of Helsinki of the World Medical Association.

The patients were enrolled at the outpatient clinic of the Internal Medicine and Nephrology department at the Faculty of Medicine, Cairo University. The study included 110 Egyptian patients, including 55 with type 2 diabetes (T2DM) and 55 with DKD diagnosed, according to the American Diabetes Association and the American Society of Nephrology. Patients recruited had to meet the following inclusion criteria: age >18 years, fasting plasma glucose (FPG) more than 7 mmol/L, postprandial glucose (PPG) exceeding 11.1 mmol/L, HbA1c ≥ 6.5%. DKD patients have an albumin-to-creatinine ratio (ACR) of more than 30 mg/g and a reduction in GFR ≤ 60 mL/min per 1.73 m^2^. ESRD stage 5 (G5) showed a further reduction in GFR <15 mL/min/1.73 m^2^.

The trial excluded patients with nephropathy due to other causes, autoimmune diseases, concurrent urinary tract infection, hepatitis, HIV positivity, glucocorticoid treatment, kidney transplantation, and cancer. Pregnancy and breastfeeding were also exclusion factors. After examining the inclusion and exclusion criteria, all participants underwent a clinical evaluation consisting of a comprehensive medical history and laboratory tests. Fifty-five healthy volunteers of the same age and gender with no history, clinical symptoms, or test results of diabetes mellitus participated in the study.

### Blood sample collection and laboratory assays

The sample size was estimated as 53 patients for each group using the G*Power v3 software with a significance level of 0.05, an effect size of 0.25, a power of 0.8, and a correlation of 0.8. Based on this assumption, a total of 165 participants in the three studied groups were considered adequate.

After 8 h fasting and 2 h following a meal, trained laboratory personnel extracted 5 mL of peripheral venous blood from each participant. The blood was collected in EDTA tubes for measuring glycosylated hemoglobin (HbA1c), frozen at -80°C until RNA extraction for circ_0000146, circ_0000072, miR-21, and miR-495 quantification, or centrifuged for 15 min at 1000 × g for plasma separation and measurement of FPG and PPG. GFR was calculated using the diet modified-kidney disease equation. Another portion of blood was kept in plain tubes and left to clot for 15 min before centrifugation at 4000 × g to collect serum. Kidney function tests (serum urea and creatinine levels), albumin, CRP, and CNR1 were determined using the serum. In addition, the ACR was calculated using two morning-collected urine samples.

### RNA extraction

Total RNA was extracted using the miRNeasy Mini Kit (Qiagen, Catalog Number: 217004, Frank furt,Germany). Quantifying and analyzing the purity of RNA samples using the NanoDrop® (ND)-1000 spectrophotometer (NanoDrop Technologies, Inc., Wilmington, USA). The total RNA yield was calculated at 260 and 280 nm using a Beckman dual spectrophotometer.

### Circ_0000146, circ_0000072, miR-21, miR-495 expression by RT-qPCR

For RT-qPCR, the TransScript® Green One-Step RT-qPCR SuperMix kit (Transgen Biotech, Cat. # AQ211-01, Beijing, China) was utilized. The protocol for determining circ_0000146 and circ_0000072 consisted of 15 min at 45°C followed by 5 min at 95°C. Subsequently, 40 cycles of PCR amplification were performed, with 15s at 95°C, 20 s at 55°C, and 30s at 72°C. Regarding miR-21 and miR-495, the following modifications were made: 94°C for 30s, then 40 cycles of 94°C for 5s, and 60°C for 30 s. Using the 2^−ΔΔCt^ method, the circRNAs and miRNAs were normalized relative to the mean Ct values of the GAPDH and RUN U6B housekeeping genes, respectively. An RT-qPCR system (StepOne, version 2.1, Applied Biosystems, Foster City, USA) was used for the analysis. The sequences of the primers are shown in Table 1.

**Table 1:** Primers’ sequences.

Gene	Primer sequence	Accession #
CNR1	F: 5’-GGCAGTGAAGAACCGATACA-3’<br>R:5’-CCCAAACCTACCAAGACAGAG-3’	NM_001160226.3
hsa_circ_0000146	F: 5’-CCACAAGCAAACCACAGTCA-3’<br>R: 5’-AATGACCACAGCCACAATGA-3’	NM_021642
hsa_circ_0000072	F: 5’-TCATGGCAATCGAGTTGAGT-3’<br>R: 5’-CAAACCAAGGAATAGCTTCCA-3’	NM_145243
hsa-miR-21	F: 5’-GTGCAGGGTCCGAGGT-3’	MIMAT000449
hsa-miR-495	F: 5’-GTGCAGGGTCCGAGGT-3’	MIMAT0002817
GAPDH	F: 5’-ACCCACTCCTCCACCTTTGA-3’<br>R: 5’-CTGTTGCTGTAGCCAAATTCGT-3’	NM_001357943.2
RUN U6B	F: 5’GGCAGCACATATACTAAAATTGGAA-3’	M14486.1
Universal reverse primer	R: 5’-GTGCAGGGTCCGAGGT-3’	N/A

### Quantitative determination of serum CRP and CNR1

The quantitative determination of CRP and CNR1 was performed using a commercially available ELISA Kit (SunLong Biotech Co., LTD, Cat. # SL0535Hu, Zhejiang, China) and (SunLong BiotechCo., LTD, Cat. # SL3387Hu, Zhejiang, China), respectively.

### Statistical analysis

The Kolmogorov-Smirnov normality test was used. We employed nonparametric tests (the Kruskal–Wallis test and the Mann–Whitney U test, *P* < 0.05) and one-way ANOVA (parametric test, *P* > 0.05). The chi-square (x^2^) test was used to analyze categorical variables. Mean and standard deviation are employed to characterize the continuous variables. Spearman’s rank correlation was employed to determine the correlation between continuous variables. IBM SPSS Statistics software version 26 was used for statistical analysis. As the cutoff value for statistical significance, a P-value of 0.05 was used. GraphPad Prism Software version 9.0.0 was used to generate the graphs.

### Bioinformatics analysis and rationale of circRNAs and miRNAs selection

Due to the lack of information regarding circRNAs and DKD, circRNAs of interest were selected using an integrated bioinformatics approach. This network was acquired in three main steps. Initially, the Gene Atlas (http://genatlas.medecine.univ-paris5.fr/) was used to identify the relevant protein-coding gene CNR1 (6q15) implicated in the pathogenesis of DKD. Second, miR-21 and miR-495 were selected because they have been identified as epigenetic regulators of the CNR1 gene (Diana tools, http://carolina.imis.athena-innovation.gr/diana_tools/web/index.php?r=tarbasev8%2Findex and TargetScan, https://targetscan.org/vert71/). HMDD v3.2 (https://www.cuilab.cn/hmddcirc_0000146) was used to examine the causality of the miRNAs and DKD. Using the CircInteractome database (https://circinteractome.nia.nih.gov/), the cirRNAs circ_0000146 and circ_0000072 were determined to be sponges for miR-21 and miR-495, respectively. Lastly, the circRNAs were examined using KEGG pathway analyses to identify target genes and likely signaling pathways associated with DKD.

## Results

### Demographic and biochemical parameters of the studied groups

No statistically significant difference was found in age or gender between the examined groups, as shown in Table 2 (*P*>0.05). The BMI of diabetic and DKD patients was significantly higher than that of the control group (*P*<0.0001). Except for calcium and phosphorus, all laboratory values were significantly higher in the DKD group than in the diabetic and control groups (*P*<0.0001).

**Table 2:** Demographic and biochemical characteristics in the studies groups. Data expressed as mean (SD) or n (%). ACR, Albumin-to-creatinine ratio; BMI, Body mass index; FPG, Fasting plasma glucose; PPG, postprandial glucose.<br>^a^ Significant from healthy controls, ^b^ Significant from diabetic patients<br>^*^ Significant at P <0.05, ^***^significant at P <0.0001

Variables	Control<br>(n=55)	Diabetic patients<br>(n=55)	DKD patients<br>(n=55)	P-value
Age (years)	51.2 (5.07)	53 (6.1)	51.2 (6.7)	0.16
Gender<br>Male<br>Female	<br>28 (50.9%)<br>27 (49.1%)	<br>19 (34.6%)<br>36 (65.4%)	<br>25(45.4%)<br>30(54.6%)	0.21
BMI	22.7 (1.56)	32.9 (5.47) ^a***^	29.1 (4.71) ^a***b*^	< 0.0001
FPG (mmol/L)	5.04 (0.31)	11.51 (3.16) ^a***^	14.46 (2.8) ^a***b*^	< 0.0001
PPG (mmol/L)	6.74 (0.43)	16.83 (3.95) ^a***^	20.1 (3.95) ^a***b*^	< 0.0001
HbA1c (%)	4.8 (0.25)	6.9 (0.35) ^a***^	7.08 (0.52) ^a***^	<0.0001
Albumin (g/L)	41.78 (3.22)	38.39 (2.3) ^a***^	36.74 (1.92) ^a***b**^	<0.0001
Urea (mmol/L)	6.4 (1.3)	6.8 (2.15)	18.6 (3.02) ^a***b***^	<0.0001
Creatinine (μmol/L)	71.5 (12.7)	80.9 (31.2)	289.7 (63.9) ^a***b***^	<0.0001
GFR (mL/min/1.73 m^2^)	97.8 (17.7)	88.8 (15.6) ^a*^	20.12 (5.8) ^a***b***^	<0.0001
ACR (mg/g)	7.8 (2.18)	20.4 (6.78) ^a***^	92.5 (72.46) ^a***b***^	<0.0001
Calcium (mmol/L)	2.2 (0.07)	2.2 (0.12)	2.3 (0.21)	0.07
Phosphorus (mmol/L)	1.13 (0.16)	1.12 (0.17)	1.13 (0.14)	0.94

### CRP and CNR1 levels among the studied groups

**Figure 1:**
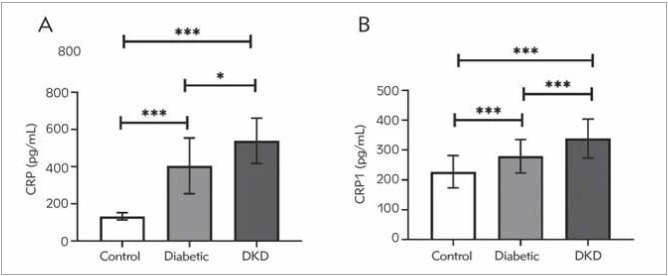
Serum CRP (A) and CNR1 (B) protein levels among studied groups. Data expressed as mean ± SD. CNR1; cannabinoid receptor protein, CRP; C-reactive protein ^*^Significant at <0.05, ^***^significant at *P*<0.0001

The serum CRP and CNR1 protein levels are depicted in Figure 1A and Figure 1B, respectively. CRP and CNR1 were significantly higher in DKD patients than in diabetic patients and healthy controls (*P*<0.0001). Moreover, diabetic patients had significantly elevated levels of CRP and CNR1 than the control group (*P*<0.0001).

### The expression levels of CNR1, circ_0000146, and 0000072, as well as miR-21 and 495 within the groups being studied

**Figure 2:**
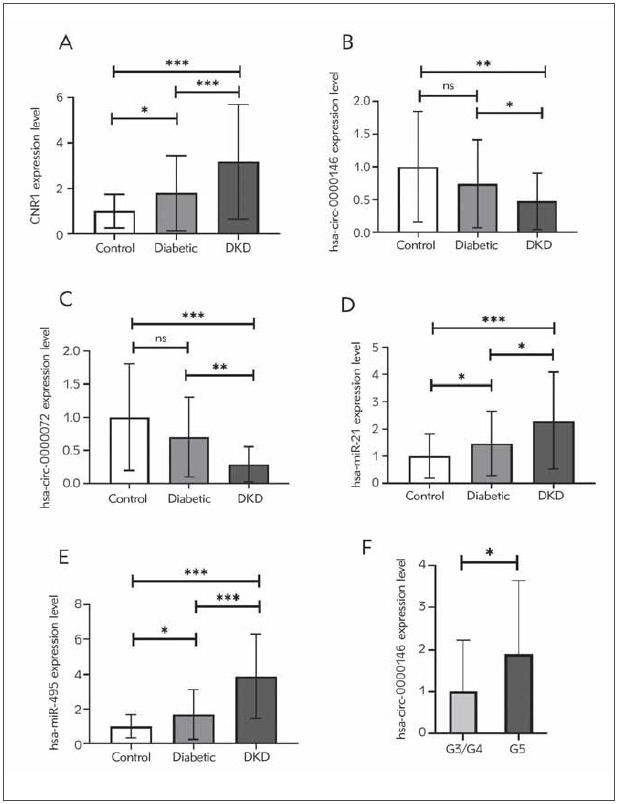
Circ_0000146 (A), circ_0000072 (B), miR-21 (C), miR-495 (D), CNR1 (E) and circ_0000146 staging (F) relative gene expression among the studied groups. Data expressed as mean ± SD. ^*^significant at *P*<0.05, ^**^significant at p<0.001, ^***^significant at *P*<0.0001

Following the ELISA results, CNR1 expression was significantly higher in DKD patients compared to both diabetic patients and the control group (*P*<0.0001). Moreover, CNR1 expression was significantly higher in diabetic patients compared to the control group at *P*<0.05 (Figure 2A).

Figure 2B, Figure 2C, Figure 2D and Figure 2E illustrated that the expression levels of circ_0000146 and 0000072 were significantly lower in DKD patients compared to patients with diabetes and the control group. While circ_0000146 and circ_0000072 demonstrated an insignificant reduction in patients with diabetes compared to the control group (*P*>0.05). In DKD patients, the expression levels of miR-21 and 495 are 1.56- and 2.3-fold higher in DKD patients compared to diabetic patients at *P* < 0.05. Moreover, compared to the control group, DKD patients displayed a 2, 3- and 3, 88-fold increase in miR-21 and 495 expression levels, respectively (*P*<0.0001). In addition, both miR-21 and 495 expression levels were elevated in patients with diabetes relative to the control group at *P*<0.05.

In addition, compared with patients with mild renal impairment, those with advanced renal impairment (G5) demonstrated a significant 1.9-fold increase in circ_0000146 (Figure 2F).

### Spearman correlations for all investigated circRNAs and miRNAs

The relationship between circ_0000146 and 0000072 with miR-21 and 495, CNR1 the inflammatory biomarker; CRP, glucose indicators; PPG, FPG, and HbA1c, and renal function predictors; ACR and GFR is provided in Table 3. circ_0000146 and 0000072 demonstrated a negative association with all estimated laboratory tests, biomarkers, and their respective target miRNAs. Circ_0000146 and 0000072 were correlated negatively with miR-21 (circ_ 0000146: r =-0.143, P=0.04, circ_0000072:r =-0.15, P=0.04) and miR-495 (circ_ 0000146: r=0.03, P=0.7, circ_0000072: r=-0.43, P<0.0001), CNR1 (circ_0000146: r =-0.19, P=0.014, circ_0000072: r=-0.2, P<0.01), the inflammatory biomarker; CRP (circ_0000146: r=-0.19, P=0.13, circ_0000072: r =-0.32, P<0.0001), blood glucose indicators; PPG (circ_0000146: r=-0.17, P=0.02, circ_0000072: r=-0.26, P 0.001), FPG (circ_0000146: r=-0.19, P=0.01, circ_0000072: r=-0.29, P<0.0001) and HbA1c (circ_0000146: r=-0.21, P=0.008, circ_0000072: r=-0.27, P<0.0001), and the renal function predictors; ACR (circ_0000146: r=-0.21, P=0.009, circ_0000072: r=-0.35, P<0.0001) and GFR (circ_0000146: r=0.2, P=0.009, circ_0000072: r=-0.29, P<0.0001).

**Table 3:** The association between circ_0000146 and 0000072 expression levels with MiRNAs, glucose indicators, inflammatory factors; CRP, CNR1, and DKD predictors; ACR and GFR. Spearman’s rho correlation test was used. R, correlation coefficient; PPG, postprandial glucose; FPG, Fasting plasma glucose; CNR1, Cannabinoid receptor type 1; CRP, C-reactive protein.

	circ_0000146		circ_0000072	
	r	P-value	r	P-value
miR-21	-0.14	0.04	-0.15	0.04
miR-495	-0.03	0.7	-0.43	<0.0001
CNR1	-0.19	0.014	-0.2	<0.01
CRP	-0.19	0.013	-0.32	<0.0001
PPG	-0.17	0.02	-0.26	0.001
FPG	-0.19	0.01	-0.29	<0.0001
HbA1c	-0.21	0.008	-0.27	<0.0001
ACR	-0.21	0.009	-0.35	<0.0001
GFR	0.2	0.009	0.29	<0.0001

### Potential diagnostic values of circ_0000146 and 0000072, hsa-miR-21, and 495 in DKD

**Figure 3:**
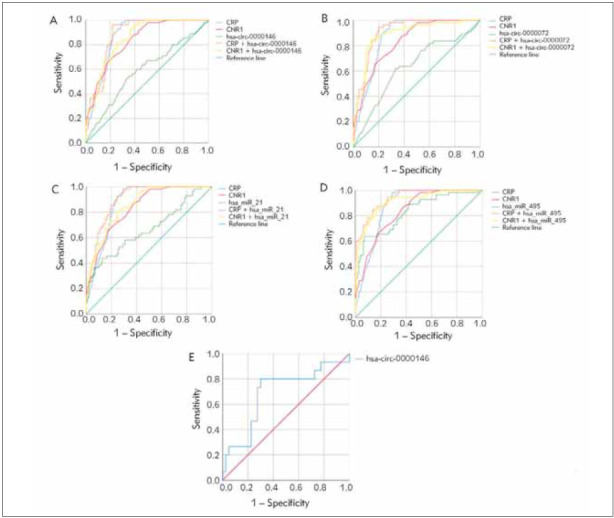
Diagnostic accuracy of circ_0000146 (A), circ_0000072 (B), miR-495 (C), has-miR-21 (D) and their combination with the DKD biomarkers; CRP and CNR1 to distinguish DKD patients and for renal staging (E) by ROC curve.

ROC curves were analyzed to evaluate the diagnostic performance of all molecules with significant differential expression. We calculated the potential diagnostic value of CNR1, CRP, circ_0000146 and 0000072, miR-495, and 21 to distinguish between patients with T2DM and DKD (Figure 3A-Figure 3D).

Circ_0000072 had an AUC value of 0.72 (sensitivity: 71%, specificity: 64%), whereas circ_0000146 had an AUC value of 0.64 (sensitivity: 71%, specificity: 64%). (AUC; 0.65, sensitivity; 63 %, specificity;62%) Compared with miR-21, miR-495 had a higher AUC value of 0.83 (sensitivity: 73%, specificity: 69%). (AUC; 0.66, sensitivity; 60%, specificity; 61%). circ_0000072 (CRP + circ_0000072: AUC;0.91, sensitivity; 91 %, specificity; 81%, CNR1 + circ_0000072: AUC;0.89, sensitivity; 87%, specificity; 81%), circ_0000146 (CRP + circ_0000146: AUC;0.88, sensitivity; 96%, specificity; 80%, CNR1 + circ_0000146: AUC; 0.85, sensitivity; 82%, specificity; 71%), miR-495 (CRP + miR-495: AUC; 0.94, sensitivity; 98%, specificity; 70%, CNR1 + miR-0000 and miR-21 (CRP + miR-21: AUC=0.9, sensitivity=98%, specificity=72%; CNR1 + miR-21: AUC=0.87, sensitivity=80%, specificity=76%). In addition, the discriminating power of circ_0000146 to distinguish between G3/G4 and G5 stages had an AUC of 0.69 (sensitivity: 73%, specificity: 72.5%, P=0.03).

### Prediction of target miRNAs of circ_0000146 and circ_0000072, and their pathway prediction analysis

Predictive software such as the CircInteractome and TargetScan databases was used to identify potential target miRNAs and genes for circ_0000146 and circ_0000072.

According to the KEGG database, the target genes of circ_0000146 were significantly involved in mitogen-activated protein kinase (MAPK), RAS, and transforming growth factor (TGF-β) signaling pathways at *P*<0.001. Alternatively, circ_0000072-targeted genes were connected to the TGF-β AMP-activated protein kinase (AMPK) and Wnt signaling pathways (Table 4).

**Table 4:** KEGG pathway enrichment analysis of circ_0000146 and circ_0000072.

KEGG signaling pathway	Genes<br>n (%)	P-value
circ_0000146		
MAPK signaling pathway	21 (5.8%)	5.4 × 10^-7^
RAS signaling pathway	16 (4.4 %)	2.8 × 10^-5^
TGF-β signaling pathway	8 (2.2 %)	1.9 × 10^-3^
circ_0000072		
TGF-β signaling pathway	14 (1.7 %)	5 × 10^-5^
AMPK signaling pathway	13 (1.6%)	1.9 × 10^-3^
Wnt signaling pathway	15 (1.8%)	4.4 × 10^-3^

## Discussion

DKD is a chronic vascular complication of T2DM leading to ESKD [12]. DKD is becoming more prevalent in developing countries and is now recognized as a worldwide health concern [13]. Consequently, the identification of noninvasive potential diagnostic and prognostic biomarkers is crucial.

In this study, CNR1 was chosen because it plays a crucial role in various pathophysiological processes that promote DKD as oxidative stress, inflammation, and fibrogenesis [14]. In the present study, the levels of CNR1 gene and protein expression were significantly higher in DKD patients in relation to others. It was reported that exposing podocytes to an increased glucose level for 48 hours caused podocyte damage and CNR1 expression [15]. CNR1 blockers were reported to normalize kidney functions and tubular injury in mice by reducing lipocalin 2, clusterin, cystatin C, and TNF expression [16]. Two neutral CNR1 receptor antagonists, AM6545 and AM4113, were previously reported to have renoprotective properties and lower kidney TGF levels [17]. CRP, an acute phase inflammatory protein, is associated with an increase in microalbuminuria and renal impairment in diabetic patients, suggesting a connection between CRP and DKD progression [5] [18]. In our study, DKD patients had significantly higher CRP protein concentrations than other participants. Dawood et al. [19] previously reported the impact of elevated serum CRP levels on the development of DKD.

CircRNAs have been reported to be associated with the onset and progression of renal diseases, including diabetic glomerular injury [9]. RT-qPCR analysis of the gene expression levels of hsa_circ_0000146 and hsa_circ_0000072 in DKD patients was exclusive to our study. We discovered that DKD patients had significantly lower hsa_circ_0000146 and hsa_circ_0000072 gene expression than diabetes patients and controls. The mechanism of action of circ_0000146 and circ_0000072 was evaluated using bioinformatics to predict their target miRNAs and genes from the CircInteractome and TargetScan databases. The predicted miRNA targets for circ_0000146 were miR-21, miR-136, miR-145, miR-217, and miR-346. The predicted targets of circ_0000072 were miR-495, miR-146, miR-136, miR-145, and miR-638. The KEGG pathway results indicated that circ 0000146’s target genes are significantly involved in the MAPK, RAS, and TGF-β signaling pathways. Alternatively, circ_0000072-targeted genes were associated with TGF-β, AMPK, and Wnt signaling pathways. MAPK signaling pathway is involved in cell signal transduction that contributes to insulin signaling and glucose transporter 4 expression levels, which are associated with insulin resistance in T2DM [20] [21]. In addition, at high glucose concentrations, p38MAPK promotes cell proliferation, protein accumulation, and TGF-β, which ultimately results in DKD [22] [23]. In addition, the Wnt signaling cascade appears to play a crucial role in regulating the development of DKD in podocyte and mesangial cell damage and kidney fibrosis [24]. Angiotensin II (AngII), the principal peptide of RAS, promotes podocyte injury and reactive oxygen species production. Its blockers can reduce progressive glomerulosclerosis [25]. MiRNAs contribute to the progression of several glomerular basement membranes and extracellular matrix alterations associated with renal tissue fibrosis [26]. Compared to diabetic patients and healthy controls, the level of miR-21 expression in DKD patients was higher. miR-21 is involved in T2DM complications due to its diverse gene regulatory functions, and its silencing ameliorates DKD [8]. MiR-21 has been reported to be overexpressed in the blood and kidney tissues of DKD patients and was correlated with ACR [27] [28]. Intriguingly, mice lacking miR-21 had lower concentrations of mesangial extension, albumin in their urine, fibrotic biomarkers, macrophage infiltration, and podocyte damage [29]. In our study, we discovered that the expression of the miR-495 gene is significantly greater in DKD patients than in diabetic patients and healthy controls. Our results concur with a previous study finding that mice injected with streptozotocin had a higher miR-495 gene expression level than their corresponding controls [30]. Moreover, miR-495 was markedly up-regulated in retinal ganglion cells treated with high glucose, increasing their apoptosis [31]. In contrast, a different study found that serum miR-495-3p levels were lower in diabetic patients with retinopathy than those without retinopathy [32].

Moreover, circ_0000146 and circ_0000072 were found to negatively correlate with miR-21 and miR-495, respectively. In addition, both circ_0000146 and circ_0000072 were negatively correlated with FPG, PPG, and glycosylated HbA1c levels. Previously, circRNA_0054633 was observed in pregnant women and associated with PPG and glycosylated hemoglobin [33]. There was also a significant negative correlation between circ_0000146 and circ_0000072 and ACR, CRP, and CNR1.

The results of the ROC curve indicated that the sensitivity and specificity of circ_0000146 and circ_0000072 alone or in combination with CRP or CNR1 were within an acceptable range and could be considered as new diagnostic biomarkers for DKD. Consequently, we may deduce that hsa_circ_0000146 and hsa_circ _0000072 may play a role in the pathogenesis of DKD, may serve as potent, novel, noninvasive biomarkers, and maybe a promising therapeutic target in DKD.

## Dodatak

### Compliance with Ethical Standards

This study was conducted in-house in accordance with the ethical standards of the World Medical Association’s Declaration of Helsinki and with an approval number: MD-83-2020. All participants gave their informed consent for this study.

### Funding

The research was funded by Al Kasr el Aini, Faculty of Medicine, Cairo university.

### Informed consent

All participants gave their informed consent for this study.

### Conflict of interest statement

All the authors declare that they have no conflict of interest in this work.
